# Artificial Intelligence-Assisted Throat Sensor Using Ionic Polymer–Metal Composite (IPMC) Material

**DOI:** 10.3390/polym13183041

**Published:** 2021-09-09

**Authors:** Jai-Hua Lee, Pei-Song Chee, Eng-Hock Lim, Chun-Hui Tan

**Affiliations:** Lee Kong Chian Faculty of Engineering and Science, Universiti Tunku Abdul Rahman, Kajang 43000, Selangor, Malaysia; xenonargon@1utar.my (J.-H.L.); limeh@utar.edu.my (E.-H.L.); tchui@utar.edu.my (C.-H.T.)

**Keywords:** ionic polymer–metal composite (IPMC), self-powered sensor, wearable sensor, support vector machine (SVM), smart sensor

## Abstract

Throat sensing has received increasing demands in recent years, especially for oropharyngeal treatment applications. The conventional videofluoroscopy (VFS) approach is limited by either exposing the patient to radiation or incurring expensive costs on sophisticated equipment as well as well-trained speech-language pathologists. Here, we propose a smart and non-invasive throat sensor that can be fabricated using an ionic polymer–metal composite (IPMC) material. Through the cation’s movement inside the IPMC material, the sensor can detect muscle movement at the throat using a self-generated signal. We have further improved the output responses of the sensor by coating it with a corrosive-resistant gold material. A support vector machine algorithm is used to train the sensor in recognizing the pattern of the throat movements, with a high accuracy of 95%. Our proposed throat sensor has revealed its potential to be used as a promising solution for smart healthcare devices, which can benefit many practical applications such as human–machine interactions, sports training, and rehabilitation.

## 1. Introduction

Oropharyngeal dysphagia is a swallowing disorder that is commonly caused by a number of neurological impairments (such as Parkinson’s disease and stroke), respiratory disorders, head and neck cancers, and genetic syndromes [1]. Without appropriate medication, it not only interferes with a patient’s quality of life but also leads to devastating effects such as dehydration, malnutrition, pneumonia, respiratory complication, or even more severe cases such as death [2]. As the current monitoring method for dysphagia patients, videofluoroscopy (VFS) can be efficient, but it requires highly trained speech-language pathologists (SLPs) to analyze the swallowing function through high-density x-ray images [3,4]. In addition, the VFS protocol requires the patient to swallow different volumes of barium bolus to enable visualization under the VFS, thereby exposing the patient to radiation [5]. Furthermore, it is not economically viable for patients with limited mobility or those who simply reside in rural areas without good resources. Therefore, developing a safe, portable, and non-invasive method to monitor the swallowing process is imperative.

One of the approaches is to attach a wearable sensor [6] somewhere around the throat to measure the bioelectrical signals emitted from the muscle during the swallowing process. Nonetheless, the current established sensor, which is usually fabricated on a rigid platform, is not suitable for the wavy surface of human skin, and it often causes low-quality signals and creates discomfort for the users. Recent advances in flexible technology can possibly alleviate this constraint by fabricating the sensor in a thin form factor [7,8,9] or using soft elastomer [10,11,12,13,14,15] as the constructive material to promote conformal contact between the sensor and the skin. For instance, a skin patch sensor is fabricated by integrating a strain gauge with a soft elastomer to provide a piezoresistive and capacitive output against laryngeal movement during swallowing [16]. The mentioned transduction principles, however, are constrained by challenges posed by the sensor’s power consumption and self-heating behavior, which may require the use of complicated power management circuitries. A self-powered piezoelectric [1,17,18] transduction mechanism that can respond well to muscle stimuli will make an excellent long-term and sustainable sensory system as it can generate an electrical output without any biasing circuit. Nonetheless, this sensing principle is only sensitive to the transient response, i.e., the earliest spike of laryngeal acceleration [19,20]. The output signal will eventually drop to zero if the muscle moves at a constant speed due to the absence of polarization in the crystal lattice of the piezoelectric material [21,22].

Noticeably, the ionic polymer–metal composite (IPMC) material is more promising for the construction of a self-powered sensor. The IPMC is an electroactive polymer that has been studied over the past few years for its outstanding sensing [23,24] and actuating performances [9,25,26,27]. The IPMC can be constructed by sandwiching a Nafion membrane (consisting of fixed anions, mobile cations, and water molecules) between two layers of metal electrodes. The pressure applied onto the IPMC material induces the mobile cations and water molecules to move from a high-stress region to a lower stress region. This ionic movement causes an imbalanced charge distribution across the membrane and forms a dielectric potential layer. The mobile cations remain in the lower-stress region until the stress is removed, thereby creating a steady-state detection. To further broaden the potential of this facile, designed material, artificial intelligence (AI) can be integrated with the sensor for advanced data interpretation.

The advancement of fifth-generation (5G) technology has propelled sensor development from a component level to a system level, where massive, real-time sensory data can be collected for more diversified applications. This new era paves the way for advanced data analytics where AI-assisted sensor technology provides the capability of decision making with respect to complex and dynamic stimuli. Machine learning (ML) is a subset of AI that enables the system to learn from the extracted data for decision making. A support vector machine (SVM), a form of supervised machine learning, has been widely applied for predicting creep behavior [28], moisture [29], etc. For instance, a 3-stage GA–SA–SVM has been used in dissolved gas analysis, with 90.36% accuracy [30]. Despite SVM involvement in numerous applications, the use of SVMs on a self-powered IPMC sensor is yet to be explored for healthcare applications.

Hence, an AI-assisted, self-powered, and flexible throat sensor is proposed herein, which has been designed and fabricated using the IPMC material for monitoring laryngeal movement. To the best of our knowledge, this is the first ever reported self-powered IPMC sensor that incorporates AI features for healthcare applications. Traditional, wearable IPMC sensors can also be applied for sitting posture monitoring [31], where good or bad sitting postures are identified based on the threshold limit. However, this straightforward recognition method may fail in a dynamic environment, where multiple muscle movements might crosstalk with the measured signal. As compared with the traditional approach, our AI-featured sensor can be used for more complex perception functions and can be operated in a dynamic environment. By attaching the IPMC sensor to the throat, the movement of the muscle can be captured by the electrical voltage output associated with the pressure exerted by the skin. To ensure good signal acquisition, we have examined the effects of using silver (a metal that is less corrosive-resistant but has higher conductivity) and gold (one that is corrosive-resistant but has lower conductivity) as materials for the electrode. The AI-assisted IPMC is able to recognize vital signals based on the vibration of the throat cords caused by activities such as swallowing, humming, nodding, and coughing actions, with an accuracy of 95%. In particular, the proposed sensing system has demonstrated a promising potential to be used for the enhancement of the intelligence and efficiency of smart healthcare systems.

This article is organized as follows. Section 2 discusses the materials and fabrication steps in the development of the IPMC sensor. Section 3 characterizes the IPMC sensor, including its temporal response, sensitivity, and machine learning recognition of swallowing, humming, nodding, and coughing actions. Section 4 concludes the overall work.

## 2. Materials and Fabrication Methods

### 2.1. Chemicals and Materials

Nafion-117 (Dupont, Wilmington, DE, USA), D+-Glucose, sodium hydroxide, silver nitrate, ammonium hydroxide, isopropanol, sodium carbonate, gold (III) chloride solution, 1, 10–phenanthroline monohydrate (Phen), and sodium sulfite were purchased from Sigma Aldrich (St. Louis, MO, USA).

### 2.2. Fabrication of Sponge-like IPMC Sensor

A sponge-like structure was formed by hot pressing sandpaper onto a Nafion-117 membrane using a hot-molding press machine (Lotus Scientific LS-22025, Shah Alam, Malaysia) under a temperature of 190 °C and a pressure of 50 kg/cm^2^ for 15 min. Then, the sandpaper was gently removed from the Nafion-117 membrane, aided by an isopropanol–water solution, produced at a mixing ratio of 1:3. Next, the Nafion-117 membrane was soaked in a 10 mM [Au(Phen)Cl_2_]Cl solution, which was synthesized according to the protocol mentioned in the literature [32], for 24 h in order to initiate Au(Phen)Cl_2_^+^ ion impregnation. The impregnated Nafion-117 membrane was then immersed in a 5 mM Na_2_SO_3_ solution for 6 h for the reduction process [31]. In the reduction process, the Au(Phen)Cl_2_^+^ ion on the surface of the Nafion-117 membrane was reduced to metallic gold, Au. The impregnation/reduction process was repeated several times until the gold electrodes were formed. The gold electroless plating was performed using the following equation in the literature [33]:

$$[\mathrm{Au}(\mathrm{Phen}){\mathrm{Cl}}_{2}{]}^{+}\;+\;{\mathrm{Na}}_{2}{\mathrm{SO}}_{3}\;+\;2{\mathrm{OH}}^{-}\;\to \;\mathrm{Au}\;+\;\mathrm{Phen}\;+\;{\mathrm{Cl}}_{2}\;+\;{\mathrm{Na}}_{2}{\mathrm{SO}}_{4}\;+\;H_{2}O$$

In order to compare the performance of the gold-coated electrode, a silver-coated electrode was fabricated according to the protocol mentioned in [23]. The silver IPMC sensor was fabricated using the silver mirror method. A Nafion-117 film with sponge-like structure was dipped into a complex [Ag(NH_3_)_2_]OH solution for the impregnation process. Next, the silver complex compound on the surface of the Nafion-117 film was reduced to metallic silver after being transferred to a D-Glucose solution for another 5 min. The silver was subsequently formed on the Nafion surface using the equation below, as found in the literature [25]. Further details on the silver mirror method can be found in our previous work [25,26,27].

$${\mathrm{CH}}_{2}\mathrm{OH}{\left(\mathrm{CHOH}\right)}_{4}\mathrm{CHO}+2\left[\mathrm{Ag}{\left({\mathrm{NH}}_{3}\right)}_{2}\right]\mathrm{OH}\to 2\mathrm{Ag}+{\mathrm{CH}}_{2}\mathrm{OH}{\left(\mathrm{CHOH}\right)}_{4}{\mathrm{COONH}}_{4}+3{\mathrm{NH}}_{3}+3H_{2}O$$

### 2.3. Characterization

The surface morphology and chemical composition of the gold-coated IPMC structure were captured and analyzed using a scanning electron microscope (SEM Hitachi, Tokyo, Japan) equipped with energy-dispersive X-ray spectroscopy (EDX). The NI ELVIS board (NI ELVIS II+, National Instrument, Austin, TX, USA) was used to capture the electrical signal from the IPMC sensor.

## 3. Results and Discussion

### 3.1. Design of the Self-Powered IPMC Sensor

**Figure 1:**
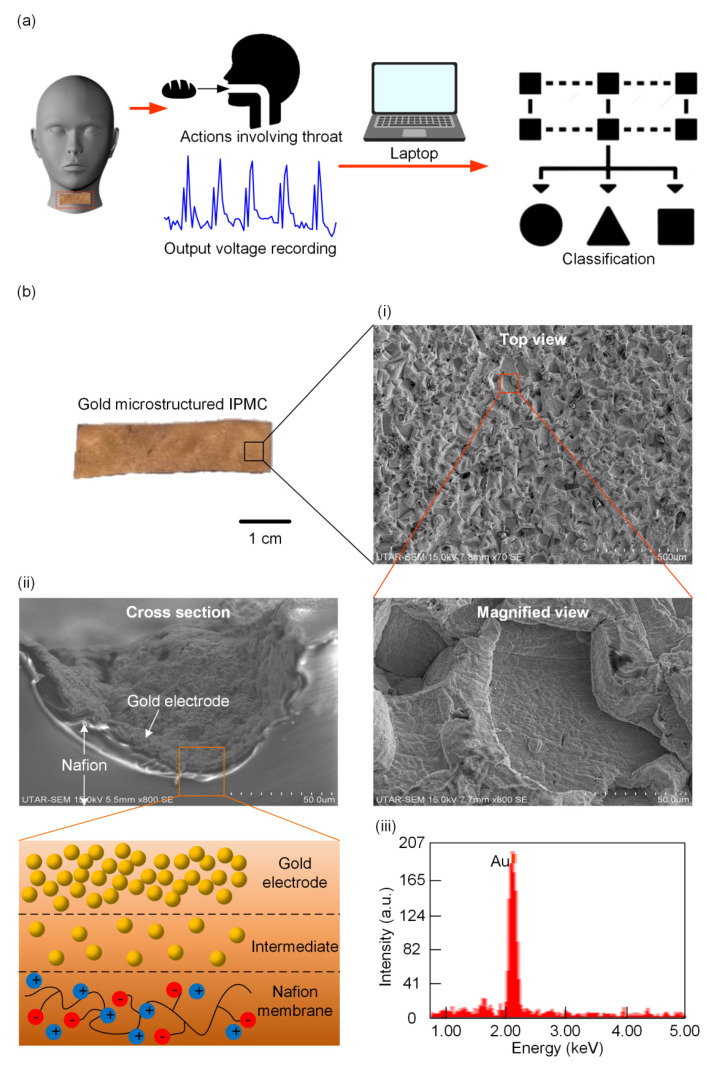
(**a**) A schematic drawing shows how the pattern recognition of the system is performed based on the input from a self-powered sensor that is attached to the throat. (**b**) Photograph of the gold-coated IPMC sensor. (**i**) SEM images examine the sponge-like surface morphology. (**ii**) SEM image and schematic illustration of the cross-sectional view of the IPMC sensor. (**iii**) EDX scanning confirms the formation of the metallic gold electrode.

Figure 1a shows the conceptual idea of mounting the IPMC sensor onto the skin for laryngeal movement monitoring. Incorporating a machine learning technique into the data analysis process enables the sensing system to recognize the pattern of throat movements, thereby offering clues for decoding the complex signals. Besides the swallowing function, the system can recognize humming, nodding, and coughing patterns. We designed our IPMC sensor with dimensions of 1 cm × 4 cm, and a thickness of 0.15 mm. This design is based on the literature finding, which investigates the effects of geometrical properties (such as thickness, shape, etc.) on sensing performance [34]. The Nafion membrane was imprinted with a sponge-like microstructure, as shown in Figure 1b(i), to improve its conformality and sensitivity. The scale bar in Figure 1b shows the dimension of the fabricated prototype. The Nafion membrane was coated with a gold (Au) material to form electrodes for data acquisition. The gold synthesis protocol was adopted from [32,35], which is a proven method for producing gold nanoparticles. The cross-sectional view of the IPMC sensor shown in Figure 1b(ii) demonstrates that the gold nanoparticles successfully covered the entire surface of the IPMC sensor, including the pores. The gold nanoparticles layer has a thickness of 2 μm. The schematic figure illustrates the three layers of the IPMC sensor. It consists of the layer of a gold electrode, an intermediate layer, and the Nafion membrane. The EDX result in Figure 1b(iii) confirms the gold formation on the sensor surface without any impurities.

### 3.2. Effect of Synthesis Conditions on the Properties of the Self-Powered IPMC Sensor

**Figure 2:**
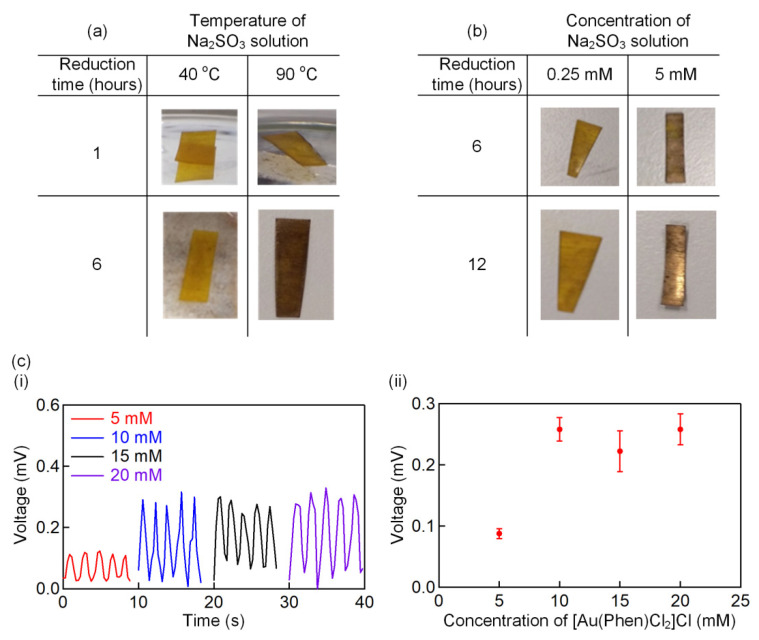
Photographs and illustrations of the (**a**) temperature and (**b**) concentration of the reducing agent, Na_2_SO_3_ solution, on the gold electrode formation. (**c**)(**i**) Temporal response of the IPMC sensor for 5 repeated cycles with different concentrations of the [Au(Phen)Cl_2_]Cl solution. (**c**)(**ii**) The steady-state output voltage behavior as a function of the concentration of the [Au(Phen)Cl_2_]Cl solution.

We conducted a study to examine the gold formation in different synthesis conditions. The formation of the gold electrode on the Nafion surface is elucidated in Figure 2a, with the reducing agent, Na_2_SO_3_ solution, heated at 40 °C and 90 °C. It can be observed that the Nafion membrane changes from a yellowish color to a dark golden color faster if a higher synthesis temperature of 90 °C is set. This can be related to the high collision rate of the molecules in the Na_2_SO_3_ solution, which stimulates a higher reaction rate [36]. The collision rate can also be enhanced by increasing the concentration [37] of the Na_2_SO_3_ solution from 0.25 mmol L^−1^ to 5 mmol L^−1^ (Figure 2b). The dark golden color on the Nafion surface turned into shinny goldish when a higher concentration of 5 mM was used. After successfully forming the gold electrode, we further quantified the IPMC sensor output by stepping up the gold complex solution, [Au(Phen)Cl_2_]Cl, from 5 mM to 20 mM. Figure 2c(i),(ii) show the temporal and steady-state responses of the IPMC sensor, respectively, when it is loaded at 30 N. It was found that the IPMC sensor fabricated with the 5 mM gold complex solution produces a considerably low voltage level. This may be caused by the high surface resistance of the sensor due to the formation of a low amount of gold nanoparticles. The sensor’s performance saturates when the concentration is increased to 10 mM, at which a further increase in concentration has no noticeable impact on the sensor’s output.

### 3.3. Characterization of the Self-Powered IPMC Sensor

**Figure 3:**
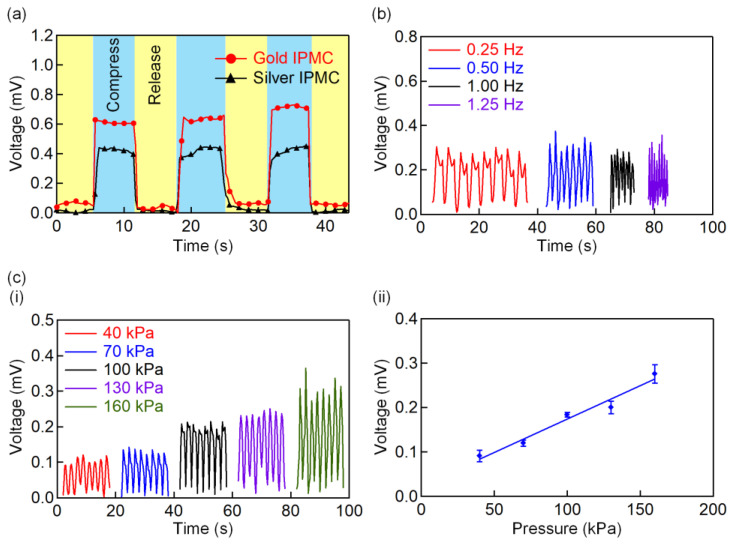
(**a**) Comparison of output voltages between the gold-coated and silver-coated IPMC sensors under three consecutive compress-and-release cycles. (**b**) Temporal response of the gold-coated IPMC sensor with 160 kPa compression pressure under different frequencies. (**c**)(**i**) Temporal response of the IPMC sensor with increased compression pressure at a constant speed of 0.5 Hz. (**c**)(**ii**) The output voltage behavior as a function of the applied pressure.

One essential path to minimize electrical noise and crosstalk contamination in the signal acquisition is to use a more corrosive-resistant metal to form the electrode. A high-conductivity metal such as silver (6.21 × 10^7^ S/m) has been reported by the literature [23] for bend-sensing and actuating [38,39] applications. Although silver has high conductivity, it is prone to corrosion. Tamagawa et al. [39] reported that silver with low corrosive-resistance could improve IPMC performance by forming a thicker electrode layer. This finding might contradict the research that has applied a corrosive-resistant metal, such as gold, to construct the electrode. Moreover, there is no comparative work on the study of the output performance of the sensor when both materials are used as the electrode. To fill this research gap, this work investigates the effects of using a metal that is more prone to corrosion but has higher conductivity, and another metal which is corrosive-resistant but has lower conductivity to form the electrode. Two IPMC sensors, one with the silver-coated sensor and the other with the gold-coated sensor (4.42 × 10^7^ S/m), were examined for their output voltages under three consecutive compression cycles. Both of the IPMC samples in Figure 3a exhibited an almost identical transient response at the same applied pressure. This result significantly proves that the IPMC material can be used as a self-powering pressure sensor. The gold-plated sensor showed a notably higher output, which is 1.5 times higher than the silver-coated electrode. This outcome proves that the corrosive-resistant property has a more dominant impact than its conductivity for sensor fabrication.

Next, Figure 3b illustrates the dynamic cyclic pressure that was performed to characterize the IPMC sensor, with a constant pressure of 160 kPa from 0.25 Hz to 1.25 Hz. At these frequencies, the outputs of the IPMC sensor remained at a constant peak-to-peak voltage (*V*_pp_ = 0.1803 mV). This behavior proves that the IPMC sensor is independent of the applied frequency. Figure 3c(i) shows the measured correlation between the output voltage and the pressure from 40 kPa to 160 kPa. Eight continuous compression cycles were acquired at the frequency of 0.5 Hz. The repetitive output patterns from the IPMC sensor for each of the pressure values demonstrated a stable and repeatable output response. It is worth noting that we did not observe any significant deterioration due to dehydration in the sensor response throughout the experiment. Nevertheless, encapsulation of an acrylic elastomer (VHB, 3M), as reported in the literature [31], was proven to be an effective way to mitigate any dehydration phenomenon. Figure 3c(ii) summarizes the output voltage of the IPMC sensor, which increases linearly (*R*^2^ = 0.9658) with the applied pressure at the sensitivity of 1.503 × 10^−6^ mV/kPa. This linear increment can be attributed to a higher stress gradient induced by the elevated pressure and initiates increased transportation of the mobile cations across the Nafion membrane.

### 3.4. Application of the IPMC Sensor for Various Throat Movement Sensing

**Figure 4:**
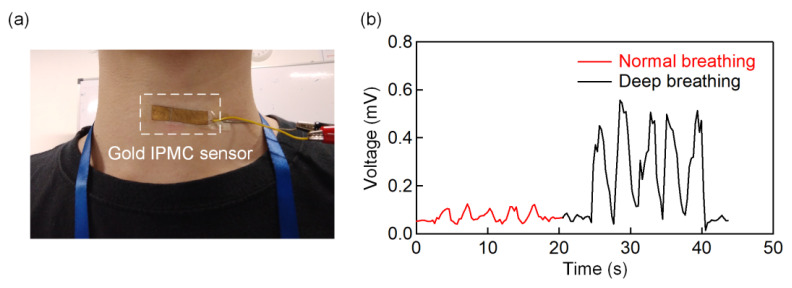
(**a**) Photograph and illustration of how the throat sensor is attached to the throat. (**b**) Output voltages from the IPMC sensor for normal breathing and deep breathing.

**Figure 5:**
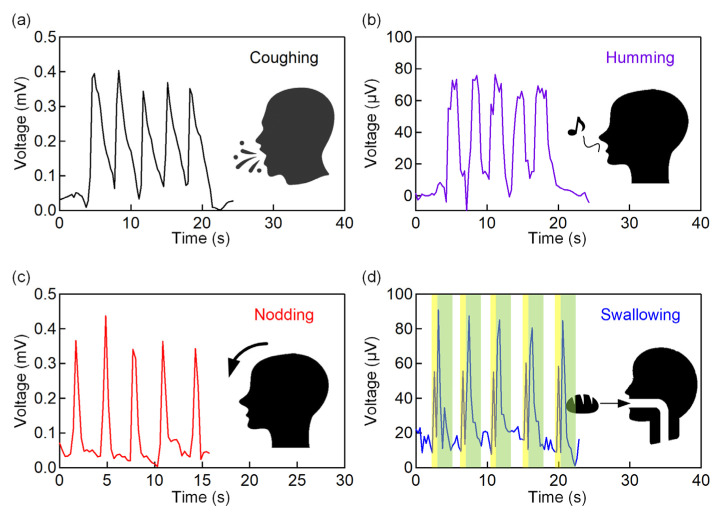
Measured output voltages from the IPMC sensor for (**a**) coughing, (**b**) humming, (**c**) nodding, and (**d**) swallowing actions.

The IPMC sensor was then attached to the throat (Figure 4a) and tested for its responses upon detection of the vital signals. We first tested the sensor responses for normal and deep breathing (Figure 4b). Each action was taken for five consecutive cycles. Inhalation and exhalation in deep breathing take in a greater volume of air and causes the throat to expand more significantly than normal breathing. As a result, the deep breathing values seen in Figure 4b demonstrated more significant amplitude changes. To show the IPMC sensor performance in detecting changes in the amplitude, we further tested the IPMC sensor for more complex operations, including the following: Figure 5a coughing, Figure 5b humming, Figure 5c nodding, Figure 5d swallowing. These actions generated different waveforms such as variations in amplitudes and speed. The coughing and nodding in Figure 5a,c pose higher amplitudes than humming and swallowing in Figure 5b,d as they involved more significant muscle movement. Compared to the temporal response in coughing (0.27 s^−1^), nodding exhibits a higher speed response (1.75 s^−1^) due to the fast movement of lowering and raising the chin [40]. However, in Figure 5d, two spikes can be observed for each swallowing cycle. The first peak (yellow highlighted section in Figure 5d) with a lower amplitude is caused by the micromotion of the hyoid before the swallowing process. After the swallowing process, the hyoid moves upwards and induces a higher amplitude spike (green highlighted section in Figure 5d) [41]. Later, the hyoid descends back to its original position and causes a decrease in IPMC voltage.

### 3.5. Data Processing via Machine Learning Technique

**Figure 6:**
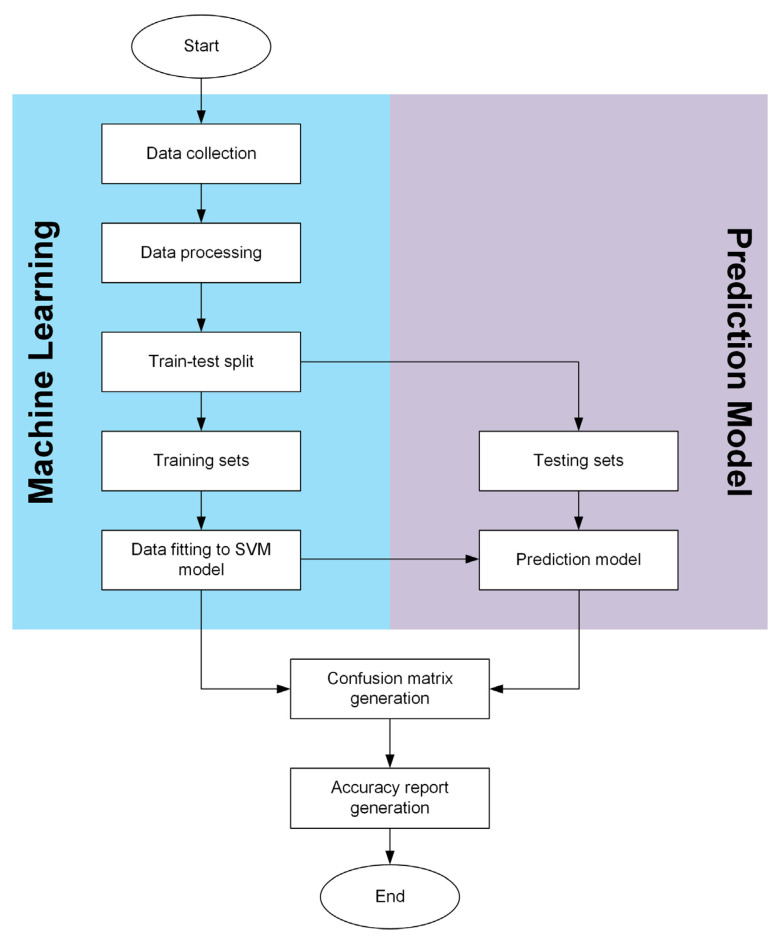
Flowchart of the SVM algorithm.

**Figure 7:**
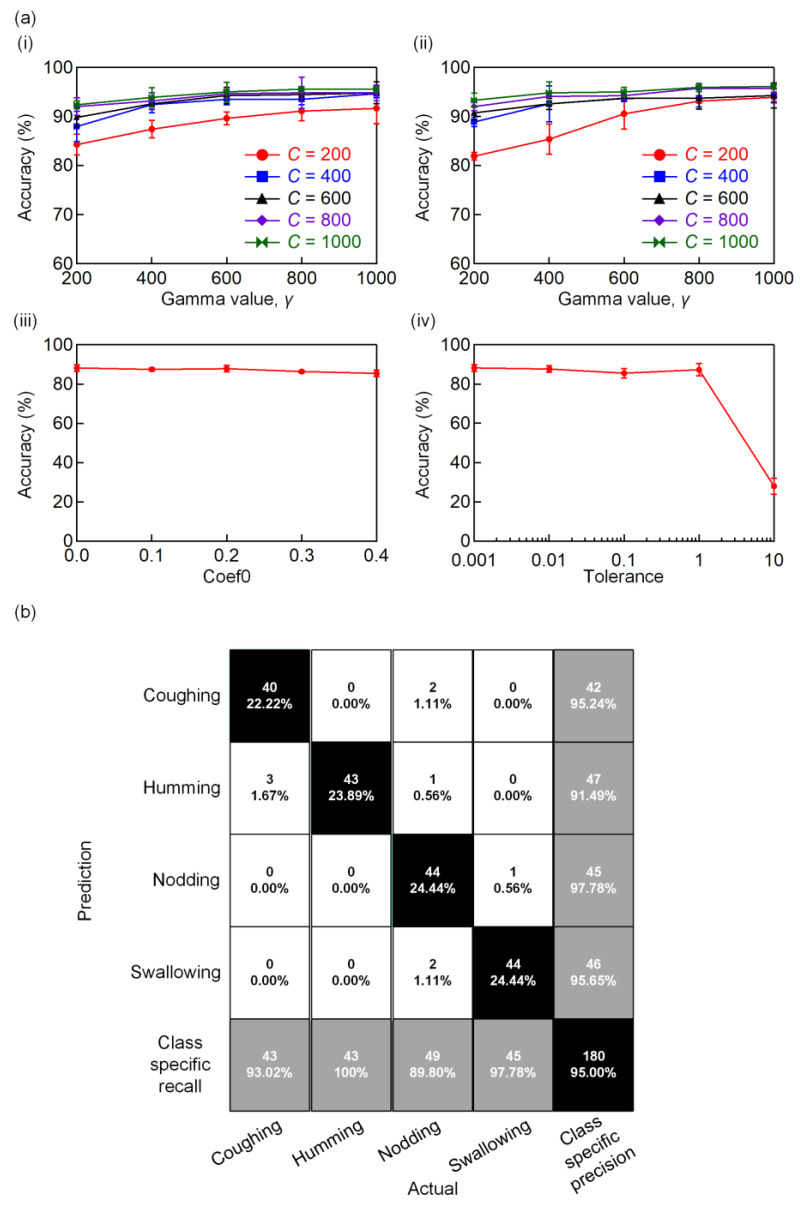
(**a**) Accuracies of the SVM models with different values of *C* and *γ* using (**i**) linear kernel and (**ii**) radial basis function kernel. (**iii**) Coef0 as a function of accuracy and (**iv**) tolerance as a function of accuracy. (**b**) Confusion matrix of the optimized SVM model.

The above results reveal that the proposed IPMC sensor is capable of perceiving complex throat movements. Machine learning is an efficient approach for handling classification automatically, where the features can be extracted from the data sets for further throat movement recognition. As compared with the conventional neural network that may require a large sample size [42], we developed an analytical algorithm (shown in Figure 6) using the support vector machine (SVM). This SVM algorithm can produce a higher accuracy even with a small sample size [42]. First, the raw voltage data in the time domain were recorded as sample features. Each action was repeated 180 times to ensure the reliability of the data set (4 actions × 180 times = 720 samples). These data were divided into 75% training datasets (540 samples) and 25% testing datasets (180 samples). The training datasets were used to train the SVM model, and the testing datasets were fed into the trained SVM model to examine the accuracy of the identification. To separate the training-set data accordingly, the kernel function was applied. Two parameters were considered in optimizing the SVM model, namely the penalty parameter, *C*, and the gamma parameter, *γ,* which were used to examine the probability of misclassification. In this work, we compared the accuracy of the recognition based on the linear kernel and radial basis function kernel, as shown in Figure 7a(i) and (ii), respectively. The optimization trials were conducted three times for each set of the parameters, and the averaged value was plotted.

The linear kernel in Figure 7a(i) achieved a higher accuracy of 95.56%, with the penalty parameter, *C* = 1000, and the gamma parameter, *γ* = 1000. A higher value for the penalty parameter, *C,* led to a smaller margin, allowing the model to classify more data correctly. The gamma parameter, *γ*, on the other hand, determines the radius of influence of a single training sample. A higher value of the gamma parameter, *γ*, allowed the hyperplane to become non-linear and achieved greater accuracy in the SVM model. We further investigated the other two parameters, Coef0 and tolerance, and their effects on the accuracy of the linear kernel. Figure 7a(iii) shows the changing trend of Coef0 as a function of accuracy. As can be seen in the figure, accuracy is independent of Coef0. Tolerance, on the other hand, resulted in a decrease in the accuracy when a higher value was used (Figure 7a(iv)). Based on the findings, we set Coef0 = 0 and tolerance = 0.001.

The optimized SVM model was then used to generate a confusion matrix, as shown in Figure 7b, from 540 training datasets. The confusion matrix provides a clearer view of the prediction performance of the SVM model. The black diagonal blocks indicate the correctly predicted actions, while the white blocks represent the incorrect predictions. The confusion matrix shows that the SVM model can assist the system in achieving a 95% accuracy of action recognition. Each action was accurately classified. For example, the swallowing action had a very high precision and recall rate of 95.65% and 97.78%, respectively. This is due to the significant double spikes feature, as seen in Figure 5d. Nevertheless, a few groups of data demonstrated a relatively lower accuracy, such as in the case of coughing. There were three outputs of coughing that were misclassified as humming. Based on Figure 5a,b, the main variation was in the amplitude, and there was a false judgement when the cough was not severe or strong enough to achieve a higher amplitude in the measured signal. Nevertheless, the confusion matrix shows that the waveforms produced by the sensor can be differentiated easily, in which each class of action has an accuracy of greater than 90%. The overall accuracy of the SVM model can be further improved by tuning the hyperparameters, for instance, *C* and *γ*. Movie S1 demonstrates the pattern recognition of the throat movement using the optimized SVM model.

## 4. Conclusions

In summary, for the first time, a self-powered IPMC sensor that can be used for throat sensing is proposed and studied. From this study, we have found that the corrosive-resistant behavior in forming the electrode has a more dominant impact than its conductivity. This hypothesis was proven when the output of the gold-coated sensor was found to be 1.5 times higher than the silver-coated sensor, although the silver material has a higher conductivity value. We have also observed that the output of the gold-coated IPMC sensor increased with the concentration of the complex solution before reaching an optimum concentration of 10 mM. Beyond the optimum concentration, the IPMC sensor did not show any noticeable improvement. The IPMC sensor was able to detect pressure and produce an output voltage of 0.09 mV to 0.27 mV with respect to the pressure range of 40 kPa to 160 kPa. It showed a high linearity output *(R*^2^ = 0.9658), with a sensitivity of 1.503 × 10^−6^ mV/kPa. Utilizing this feature, we further expanded the capability of the IPMC sensor for realizing advanced throat movement recognition. The self-powered IPMC sensor was able to distinguish different pressures exerted by throat movements. Based on the amplitude and speed of the throat movement, the optimized SVM model was able to recognize coughing, humming, swallowing, and nodding actions at a high accuracy of 95%. The enhanced intelligence of the IPMC sensor can be further applied to the internet of things (IoT) in a human–machine interaction platform for an improved human lifestyle.

## Data Availability

Data are contained within the article.

## Supplementary Materials

The following are available online at https://www.mdpi.com/article/10.3390/polym13183041/s1, Movie S1: Artificial Intelligence-Assisted Throat Sensor Using Ionic Polymer–Metal Composite (IPMC) Material.

## References

[1] Iizuka M., Kobayashi M., Hasegawa Y., Tomita K., Takeshima R., Izumizaki M. (2018). A new flexible piezoelectric pressure sensor array for the noninvasive detection of laryngeal movement during swallowing. J. Physiol. Sci..

[2] Rommel N., Hamdy S. (2016). Oropharyngeal dysphagia: Manifestations and diagnosis. Nat. Rev. Gastroenterol. Hepatol..

[3] Kelly A., Leslie P., Beale T., Payten C., Drinnan M. (2006). Fibreoptic endoscopic evaluation of swallowing and videofluoroscopy: Does examination type influence perception of pharyngeal residue severity?. Clin. Otolaryngol..

[4] Logemann J.A., Rademaker A.W., Pauloski B.R., Ohmae Y., Kahrilas P.J. (1998). Normal swallowing physiology as viewed by videofluoroscopy and videoendoscopy. Folia Phoniatr. Et Logop..

[5] Fattori B., Giusti P., Mancini V., Grosso M., Barillari M., Bastiani L., Molinaro S., Nacci A. (2016). Comparison between videofluoroscopy, fiberoptic endoscopy and scintigraphy for diagnosis of oro-pharyngeal dysphagia. Acta Otorhinolaryngol. Ital..

[6] Shieh W.Y., Wang C.M., Chang C.S. (2015). Development of a portable non-invasive swallowing and respiration assessment device. Sensors.

[7] Dinh T., Phan H.P., Dao D.V., Woodfield P., Qamar A., Nguyen N.T. (2015). Graphite on paper as material for sensitive thermoresistive sensors. J. Mater. Chem. C.

[8] Dinh T., Phan H.P., Qamar A., Woodfield P., Nguyen N.T., Dao D.V. (2017). Thermoresistive effect for advanced thermal sensors: Fundamentals, design considerations, and applications. J. Microelectromech. Syst..

[9] Chang X.L., Chee P.S., Lim E.H., Tan R.C.C. (2019). A novel crenellated ionic polymer-metal composite (IPMC) actuator with enhanced electromechanical performances. Smart Mater. Struct..

[10] Chee P.S., Arsat R., Adam T., Hashim U., Rahim R.A., Leow P.L. (2012). Modular architecture of a non-contact pinch actuation micropump. Sensors.

[11] Rusli M., Chee P.S., Arsat R., Lau K.X., Leow P.L. (2018). Electromagnetic actuation dual-chamber bidirectional flow micropump. Sens. Actuators A.

[12] Chong Y.S., Yeoh K.H., Leow P.L., Chee P.S. (2018). Piezoresistive strain sensor array using polydimethylsiloxane-based conducting nanocomposites for electronic skin application. Sens. Rev..

[13] Low J.H., Chee P.S., Lim E.H. (2019). Deformable liquid metal patch antenna for air pressure detection. IEEE Sens. J..

[14] Low J.H., Chee P.S., Lim E.H., Lee K.Y. (2020). Compact organic liquid dielectric resonator antenna for air pressure sensing using soft material. Sci. Rep..

[15] Low J.H., Chee P.S., Lim E.H., Ganesan V. (2020). Design of a wireless smart insole using stretchable microfluidic sensor for gait monitoring. Smart Mater. Struct..

[16] Kim M.K., Kantarcigil C., Kim B., Baruah R.K., Maity S., Park Y., Kim K., Lee S., Malandraki J.B., Avlani S. (2019). Flexible submental sensor patch with remote monitoring controls for management of oropharyngeal swallowing disorders. Sci. Adv..

[17] Ertekin C., Pehlivan M., Aydoǧdu I., Ertaşl M., Uludaǧ B., Çlelebi G., Çlolakoǧlu Z., Saǧduyu A., Yüceyar N. (1995). An electrophysiological investigation of deglutition in man. Muscle Nerve.

[18] Ertekin C., Yüceyar N., Karasoy H. (2001). Electrophysiological evaluation of oropharyngeal swallowing in myotonic dystrophy. J. Neurol. Neurosurg. Psychiatry.

[19] Tsuga K., Hayashi R., Sato Y., Akagawa Y. (2003). Handy measurement for tongue motion and coordination with laryngeal elevation at swallowing. J. Oral Rehabil..

[20] Abe S., Kaneko H., Nakamura Y., Watanabe Y., Shintani M., Hashimoto M., Yamane G., Ide Y., Shimono M., Ishikawa T. (2002). Experimental device for detecting laryngeal movement during swallowing. Bull. Tokyo Dent. Coll..

[21] Han B., Yu X., Ou J. (2014). Self-sensing Concrete in Smart Structures.

[22] Tiwana M.I., Redmond S.J., Lovell N.H. (2012). A review of tactile sensing technologies with applications in biomedical engineering. Sens. Actuators A.

[23] Lee J.H., Chee P.S., Lim E.H. Development of a self-powered and stretchable sensor for wearable electronics. Proceedings of the 2020 IEEE-EMBS Conference on Biomedical Engineering and Sciences (IECBES).

[24] Low J.H., Chee P.S., Lim E.H., Ganesan V. Kirigami-structured and self-powered pressure sensor using electroactive polymer. Proceedings of the 2021 IEEE 34th International Conference on Micro Electro Mechanical Systems (MEMS).

[25] Chang X.L., Chee P.S., Lim E.H., Chong W.C. (2018). Radio-frequency enabled ionic polymer metal composite (IPMC) actuator for drug release application. Smart Mater. Struct..

[26] Cheong H.R., Teo C.Y., Leow P.L., Lai K.C., Chee P.S. (2018). Wireless-powered electroactive soft microgripper. Smart Mater. Struct..

[27] Cheong H.R., Nguyen N.T., Khaw M.K., Teoh B.Y., Chee P.S. (2018). Wirelessly activated device with an integrated ionic polymer metal composite (IPMC) cantilever valve for targeted drug delivery. Lab Chip.

[28] Yang C., Ma W., Zhong J., Zhang Z. (2021). Comparative study of machine learning approaches for predicting creep behavior of polyurethane elastomer. Polymers.

[29] Zhang Y., Li J., Fan X., Liu J., Zhang H. (2020). Moisture prediction of transformer oil-immersed polymer insulation by applying a support vector machine combined with a genetic algorithm. Polymers.

[30] Huang X., Zhang Y., Liu J., Zheng H., Wang K. (2018). A novel fault diagnosis system on polymer insulation of power transformers based on 3-stage GA–SA–SVM OFC selection and ABC–SVM classifier. Polymers.

[31] Liu Y., Hu Y., Zhao J., Wu G., Tao X., Chen W. (2016). Self-powered piezoionic strain sensor toward the monitoring of human activities. Small.

[32] Harris C. (1959). 136. Nitrogenous chelate complexes of transition metals. Part I. The constitution and properties of the 1: 10-phenanthroline complexes of tervalent gold. J. Chem. Soc..

[33] Park I.S., Kim S.M., Kim D., Kim K.J. The mechanical properties of ionic polymer-metal composites. Proceedings of the Electroactive Polymer Actuators and Devices (EAPAD) 2007.

[34] Wang J., Wang Y., Zhu Z., Wang J., He Q., Luo M. (2019). The effects of dimensions on the deformation sensing performance of ionic polymer-metal composites. Sensors.

[35] Byun J.M., Hwang T., Kim K.J. (2019). Formation of a gold nanoparticle layer for the electrodes of ionic polymer–metal composites by electroless deposition process. Appl. Surf. Sci..

[36] Arrhenius S. (1889). Über die Reaktionsgeschwindigkeit bei der Inversion von Rohrzucker durch Säuren. Z. Phys. Chem..

[37] Mickey C.D. (1980). Chemical kinetics: Reaction rates. J. Chem. Educ..

[38] Chung C.K., Fung P., Hong Y., Ju M.S., Lin C.C.K., Wu T. (2006). A novel fabrication of ionic polymer-metal composites (IPMC) actuator with silver nano-powders. Sens. Actuators B Chem..

[39] Tamagawa H., Okada K., Mulembo T., Sasaki M., Naito K., Nagai G., Nitta T., Yew K.C., Ikeda K. (2019). Simultaneous enhancement of bending and blocking force of an ionic polymer-metal composite (IPMC) by the active use of its material characteristics change. Actuators.

[40] Kihara H., Fukushima S., Naemura T. Analysis of human nodding behavior during group work for designing nodding robots. Proceedings of the Proceedings of the 19th International Conference on Supporting Group Work.

[41] Li Q., Minagi Y., Hori K., Kondoh J., Fujiwara S., Tamine K., Inoue M., Maeda Y., Chen Y., Ono T. (2015). Coordination in oro-pharyngeal biomechanics during human swallowing. Physiol. Behav..

[42] Jiang X., Chen R., Zhu H. (2021). Recent progress in wearable tactile sensors combined with algorithms based on machine learning and signal processing. APL Mater..

